# MicroRNA Profiling: From Dark Matter to White Matter, or Identifying New Players in Neurobiology

**DOI:** 10.1100/tsw.2007.201

**Published:** 2007-11-02

**Authors:** Anna M. Krichevsky

**Affiliations:** Center for Neurologic Diseases, Brigham and Women's Hospital and Harvard Medical School, Boston, MA 02115, USA

**Keywords:** microRNA, qRT-PCR, expression profiling, brain

## Abstract

Contemporary biology has been revolutionized by a recently discovered class of small regulatory RNA molecules, microRNAs (miRNAs). Missed by researchers for decades due to their tiny size, usually mapping to non-protein-coding regions of genomes, miRNAs and miRNA-mediated regulatory networks have been the “dark matter” of molecular biology. Deciphering miRNA pathways and functions in the CNS of complex organisms is tightly linked to understanding miRNA expression patterns. To facilitate these emerging studies, I here review the basic principles of medium- and high-throughput technologies available for miRNA expression profiling.

## INTRODUCTION

MicroRNAs (miRNAs) are a novel class of small nonprotein-coding RNA molecules that negatively regulate gene expression in various organisms. Longer RNA precursors of miRNAs are transcribed from cellular genomes of plants and animals, and undergo sequential processing by several RNases III to produce small (18–25 nt) mature miRNA regulators. The mature miRNAs are then incorporated into the RNA-induced silencing complex (RISC). By targeting the mRNA of protein-coding genes for either cleavage or translational repression, miRNAs play a critical role in a variety of normal biological processes, e.g., development, control of cell growth, proliferation, lineage determination, and metabolism[1,2]. In the past few years, miRNAs have also been implicated in the etiology of a variety of complex diseases, including Tourette’s syndrome[3], Fragile X syndrome[4], and especially in cancers of various origins[5], including brain tumors[6,7].

In animal and human cells, miRNAs share only partial complementarity to their mRNA targets, and the extent of miRNA-mRNA base pairing, as well as other conditions required for the targeting, are not yet fully established. Multiple studies indicate the importance of the 5’ end of the miRNA (first 2–8 nt, called “seed”) for proper loading into RISC, and subsequently for the miRNA targeting function[8,9]. Therefore, most computational algorithms designed for target predictions use a miRNA seed to search for complementarity in the 3’ UTR of mRNAs. This approach, however, misses many targets as there are other variables, mostly unknown, that dictate miRNA regulation. Molecular determinants of miRNA-

directed mRNA cleavage vs. translational repression so far are also poorly defined. Generally, there is a good correlation between the degree of complementarity and the probability of mRNA cleavage. When a miRNA guides cleavage, the cut is at precisely the same site as that seen for siRNA-mediated cleavage, i.e., between the nucleotides pairing to residues 10 and 11 of the miRNA[10,11]. The majority of characterized mRNA targets are regulated through the binding sites within their 3' UTR regions. However, recent results suggest that association with any position on a target mRNA is mechanistically sufficient for a miRNA-mediated RISC to exert repression of translation at some step downstream of initiation[12].

While less than 1000 miRNAs have been cloned so far, bioinformatics predicts that many thousand miRNAs may be encoded in the human genome[13]. The experimental data accumulated during recent years and computational algorithms suggest that each miRNA may potentially regulate numerous (tens to hundreds) mRNAs[8,14]. Conversely, one mRNA transcript can be potentially regulated by multiple miRNAs, including miRNAs with identical seed sequence (that can be classified as family members), and unrelated miRNAs that target different sites within the message[15]. Taken collectively, these data indicate that expression of at least 20–30% of human protein-coding genes is modulated by miRNAs[8,16]. Given the central role of miRNA regulation in a cell and its wide-ranging influence on gene expression, it is surprising that relatively little work has been reported to date regarding miRNA impact in the mammalian CNS.

Table 1 presents a list of miRNAs with functions described in developing or mature mammalian CNS. Several reviews give detailed overviews of the field[17,18,19]. The major goal of this paper, however, is to give a quick introduction to miRNA work to those neuroscientists who are about to join this fascinating journey. While the general approach and experimental design may vary depending on a specific question raised by an investigator, in most cases, the first step will be a screen of miRNA molecules expressed in a system of interest, or differentially expressed under certain conditions or treatments. In this essay, I will focus on the principles behind techniques for medium- or high-throughput analysis of miRNA expression patterns.

## MICRORNA ARRAYS

In recent years, many tools have been developed to facilitate miRNA research. There are at least 450 distinct miRNA species expressed in the mammalian brain. Therefore, technologies for their high-throughput profiling are clearly needed. Just 5 years ago, many considered simultaneous detection of numerous small RNA molecules to be impossible. Since miRNA molecules are tiny, even small variations in their length and GC nucleotide content results in a principal difference in their biochemical properties, particularly the melting temperature (Tm) of miRNA in a hybridization reaction. In fact, a 20-nt RNA molecule with 20% GC content will have delta Tm of 29 degrees compared to a 24-nt molecule with 70% GC (40 vs. 69$^{o}$C). The small size of miRNAs provides very little space for design, optimization, and labeling probes. Therefore, principal modifications of conventional hybridization-based high-throughput methods (e.g., arrays) are required for simultaneous detection of multiple miRNAs. New approaches for miRNA isolation and labeling, oligonucleotide probe design, array printing, and performance have been developed and used by a number of laboratories. Several companies offer products (e.g., preprinted miRNA arrays, probe sets, and kits for miRNA purification and labeling) and services for miRNA array profiling. I will summarize here the key principles of miRNA arrays; for more detailed analysis of arrays and, particularly, issues associated with normalization of the arrays, see Davison et al.[20].

**TABLE 1**
**miRNAs with Potential Functions in Developing and Mature Mammalian Nervous System and Human Brain Diseases**

| miRNA | Organism/System | Function | mRNA Target | Ref. |
|---|---|---|---|---|
| miR-196a | Mouse embryogenesis | Potentially involved in neural patterning | HoxB8 | [60,61] |
| miR-124a | Human HeLa cells; rodent spinal cord development | Negative regulation of neural progenitor and non-neuronal mRNAs | Multiple; laminin gamma 1, integrin beta1, SCP1 | [14,62,63] |
| miR-124a and miR-9 | Mouse ES cell-derived neurogensis | Affect neuron to glia ratio during neural lineage differentiation | Unknown | [33] |
| miR-134 | Rat hippocampal neurons | Controls development of dendritic spines | Limk1 | [64] |
| miR-132 | Rat cortical neurons; mouse suprachiasmatic nucleus | Induces neurite outgrowth; modulates light-induced clock resetting | P250GAP; RFX4 | [65,66] |
| miR-219 | Mouse suprachiasmatic nucleus | Regulates circadian period length | SCOP | [66] |
| miR-21 | Human glioma | Antiapoptotic | Unknown | [6] |
| miR-9, miR-125a, miR-125b | Human neuroblastoma cells | Controls proliferation of human neuroblastoma cells | Tropomyosin-related kinase C | [67] |

## RNA Purification

The first factor critical for obtaining reproducible miRNA array profiles is the quality of the RNA samples. Most of the RNA isolation methods developed in the past were originally designed for studies of longer, protein-coding mRNA and are, therefore, not always suitable for miRNA work. Commonly used extraction methods, for example, usually employ RNA concentration by alcohol precipitation as one of their final steps, which does not quantitatively recover small RNA molecules. Our preferred method of choice is a mirVANA miRNA isolation kit. For half-quantitative miRNA analysis, when significant amounts of initial cell or tissue material are available (equivalent of at least several micrograms of total RNA), Trizol/Tri-reagent also works reasonable well.

## Enrichment miRNAs exist in three forms: short mature miRNA, hairpin pre-miRNA, and long pri-miRNA[21]. Since only the mature miRNA is active, it is important to eliminate array signals from the pre- and pri-miRNAs. This problem can be addressed by further purification or enrichment of a small RNA fraction. Furthermore, enrichment of the RNA sample with small molecules increases the array specificity by reducing cross-hybridization with abundant longer molecules (rRNA, tRNA, mRNA, etc.). Polyacrylamide gel electrophoresis (PAGE) has been routinely employed for size selection of small RNAs. However, this procedure is not very reproducible and efficient. We have successfully used Amicon YM-100 columns to rapidly filtrate small RNA molecules from the total RNA[22]. This method allows very efficient removal of almost all RNA molecules >70 nt. Ambion, Inc. has developed a fractionation flashPAGE system to purify miRNA fraction with an 80% yield[23].

## Labeling

As has been mentioned earlier, the inherently small size of miRNAs limits the choice of labeling techniques relative to those conventionally used for the labeling of longer mRNAs. Mature miRNAs are single-stranded RNAs that are 5’ phosphorylated and 3’ hydroxylated, and these properties should be taken into account for appending a label. Furthermore, miRNAs represent only a small fraction (~0.01%) of the mass of a total RNA sample, making it very important to label them with the highest specific activity possible. Several different protocols have been used for direct miRNA labeling, including end-labeling with ATP by T4 polynucleotide kinase[22], ligation of labeled dinucleotides by T4 RNA ligase[24], tailing by poly(A) polymerase[23], and on-slide RNA-primed Klenow extension[25]. Each one of these methods has its advantages. For example, ligation-based labeling uses 3’ OH groups of mature miRNA as substrates and, therefore, enables us to omit a step of small RNA purification/enrichments. However, RNA ligases are prone to bias because the enzyme kinetics is sequence dependent, thus possibly producing inaccurate representation of the miRNAs present in a target pool. 5’ Radiolabeling with $\gamma^{33}$P ATP in the phosphorylation reaction with T4 polynucleotide kinase significantly improves the array sensitivity. Most commercial labeling kits use fluorescence for the labeling, however. In general, 5–10 μg of total RNA is sufficient to detect a large portion of directly labeled miRNAs. RT-PCR–based amplification of the miRNA fraction can dramatically increase sensitivity and has been employed by several groups[26,27,28,29]. However, this method requires a number of enzymatic steps and may also introduce an amplification bias in the miRNA representation in a sample. We, therefore, prefer direct labeling as the simplest and most representative labeling method.

## Platforms and Probes

The most common custom miRNA array platform consists of antisense DNA oligonucleotide probes that are designed to hybridize with respective mature miRNAs, and spotted or printed onto glass slides or nylon membranes. In principle, all probes should have an equal Tm of hybridization with their target miRNAs to achieve a uniform hybridization and accurate signal representation for all tested miRNAs, The length (18–25 nt) and GC content (20–70%) of miRNA molecules, however, have a major impact on the Tm of their hybridization with full-length probes. The following probe design strategies can be employed to narrow the distribution of Tms on an array and, thus, to allow tighter control of specificity during samples hybridization[29]. First, a specific Tm (typically, 54 or 55$^{o}$C) that is the predicted Tm for most miRNAs on the array is chosen and designated as the Tm of the array (TmA). Probes with calculated Tms exceeding TmA are then truncated to bring their Tm to TmA, while retaining the segment that affords the greatest discrimination among known miRNAs. The few remaining probes with a calculated Tm lower than TmA are elongated with several additional nucleotides on their 3’ ends to extend complementarities into sequences of pre-miRNAs. An alternative strategy is to use longer, 30–40 nt, probes corresponding to pre-miRNAs, while including the sequences complementary to mature miRNAs. This allows a little more sequence space to design all probes with a uniform Tm. In addition to probes for miRNAs, multiple control oligonucleotides with two to three mismatches are usually spotted to the array in order to estimate specificity of hybridization conditions.

Chemically modified probes can be utilized to increase both the specificity and sensitivity of miRNA arrays. LNA oligonucleotides (LNA is a synthetic RNA/DNA analog), for example, are characterized by an increased thermostability of duplex formed with a miRNA. The biophysical properties of LNA can be exploited to design probe sets that provide uniform, high-affinity hybridizations yielding superior detection sensitivity, and that make it possible to discriminate between a few nucleotide differences and, therefore, between closely related miRNA family members[30]. Unfortunately, LNA oligonucleotides significantly increase the price of the chip, thereby limiting the number of samples one can afford to profile.

We found that the concatamerization of DNA probes significantly enhances an array's signal. We have also found that trimeric probes are optimal in terms of signal-to-noise ratio, accuracy of DNA synthesis, and cost of the probe set. For example, we synthesize a triple repeat antisense sequence to obtain a 63-mer probe for 21-nt miRNA. Such probes are spotted with pin-replicator and immobilized onto GeneScreen Plus nylon membranes, followed by array hybridizations with 5' end-labeled small RNA[22]. In combination with radiolabeling, these arrays are at least as sensitive as LNA-based arrays, while their cost is substantially lower. Hundreds of membranes can be printed in house and, since they are reusable, employed for miRNA profiling of thousands of samples[6,22,31,32,33]. The spot signal intensity on the membranes is analyzed using conventional autoradiography and phosphoimaging. The technique, therefore, does not require any special equipment. The arrays are expandable with probes for newly discovered miRNAs; the last version of our chip includes probes for ~500 human miRNAs.

## Analysis of Array Data

In principle, miRNA arrays are analyzed as they are by better-developed mRNA microarrays. Davison et al.[20] have thoroughly addressed an impact of analysis methods in general and normalization techniques in particular. The most popular normalization approach involves the preprocessing of data by averaging of technical replicates, and background subtraction followed by median normalization and logarithmic transformation. Since the biology of miRNA is just starting to unravel, and given that current miRNA arrays are much smaller than mRNA arrays (designed for ~400–1000 genes vs. ~40,000 genes), several important differences should be noted. First, there is presently no way to quantify the tiny amount of miRNA in a sample of total RNA (typically ~0.01%). The fundamental assumption that the same amount of miRNA is extracted from a given amount of total RNA may, therefore, be invalid. Several studies suggest, in fact, that the total amount of miRNA is reduced in some cell types during development and in certain cancers[34,35]. One interesting example is T-lymphocyte development where the miRNA expression has been directly quantified. The calculated miRNA pool is highly dynamic throughout T-lymphocyte development, ranging from 5000 copies per cell in DP thymocytes to 33,000 copies per cell in DN4 thymocytes. Although the estimated size of the miRNA pools covaried closely with changes in total RNA level at the various stages of T-cell development, the calculated miRNAs:total RNA ratio changes by a factor of two during T-lymphocyte development. This should be remembered if scaling and normalization of the array data for "total signal" are applied. Second, most normalization methods developed for mRNA microarrays rely heavily on the assumption that a very small fraction of genes is changing significantly between samples. Third, roughly equal numbers of mRNA genes are usually up- and down-regulated among the samples. Both statements are incorrect for miRNA expression profiling, for instance, of developmental processes, in which multiple miRNAs are up-regulated and very few are down-regulated during certain time intervals. Finally, all present miRNA chip platforms are likely to be incomplete since the discovery of novel miRNAs is still in progress. Although it seems that a majority of relatively abundant miRNAs have been already cloned and are listed in the databases, we cannot rule out that specific conditions may trigger the expression of new and unknown small RNA molecules. Theoretically, such incomplete coverage of the miRNA chip may also skew the data analysis, especially when very different biological conditions (normal vs. disease) are compared.

## Validation Steps

Multiple "unknowns" mentioned above bring us to the issue of array validation. None of the array techniques provide absolute specificity to distinguish reliably between hybridizing sequences that have only one or a few nucleotide mismatches. Therefore, the arrays cannot efficiently discriminate between close miRNA paralogs. This limitation is alleviated somewhat by the fact that for most miRNAs, the most closely related paralogs differ by three mismatches or more. It is presently difficult to estimate the degree of functional difference between those miRNA paralogs and, therefore, how important a variation of expression of individual family members might be. There are publications suggesting that single/few nucleotide changes may effect miRNA localization in the cell and its mRNA targeting potential[36,37], making it important to discriminate between the expression of the close paralogs. In light of this idea, as well as variable effects of normalization on miRNA array data readouts, we would like to emphasize the need for quantitative and specific validation assays, such as qRT-PCR or Northern blot, in order to corroborate conclusions derived from miRNA arrays.

miRNA arrays have been widely used for profiling normal animal and human tissues, as well as in human diseases. Importantly, miRNA profiling in some cases, like several types of cancers, appears to be more indicative than mRNA profiling. Applying bead-based flow cytometric miRNA expression profiling, Lu et al.[34] were able to classify poorly differentiated tumors successfully, whereas mRNA profiles were highly inaccurate when applied to the same samples. Using home-made arrays, we can classify various brain tumors and grade human gliomas with high accuracy (the data to be published elsewhere). Recent array profiling implicated miRNA in schizophrenia by identifying 16 dysregulated miRNAs in postmortem prefrontal cortex from schizophrenia patients[38]. Given that the results of mRNA expression analyses in psychiatric fields have been notoriously discordant, future studies are necessary to validate miRNAs as biomarkers and probably etiologic factors in human neurologic and psychiatric disorders.

## QUANTITATIVE (REAL-TIME) RT-PCR METHODS

Many important biological questions require single or few cell resolution approaches. This is especially true for CNS biology since the enormous complexity of brain structure and activity is achieved through operations of numerous molecular mechanisms controlling millions of diverse cellular interactions and events that sometimes must be dissected and studied individually. Clearly, array hybridization methods that require relatively large amounts (micrograms) of RNA are not sufficiently sensitive to profile miRNA expression in a single cell or few cells. Theoretically, quantitative RT-PCR (qRT-PCR) amplification of miRNA can provide a more sensitive method of profiling. The reaction was first adapted for quantification of longer miRNA precursor molecules[39], however, their expression does not always correlate directly with levels of functional mature miRNAs. Since mature miRNAs are only 18–23 nt, clever modifications have been required to develop qRT-PCR techniques for their quantification.

Two different quantitative real-time PCR methods have been reported and found useful in commercially developed products. In the first of them, total RNA, including miRNA, is polyadenylated and reverse transcribed with a poly(T)-adapter primer. The cDNA is then amplified in a real-time PCR using the miRNA-specific forward primer and the sequence complementary to the poly(T) adapter as the reverse primer[40]. The second method is based on use of stem-loop reverse primers with short protruding sequences that are cognate to 3’ end of miRNA to prime the RT reaction[41]. The next step of the reaction is TaqMan PCR amplification with loop sequences of the original reverse primers and miRNA-specific forward primers and TaqMan probes (Fig. 1). The idea here is multifold. First, the stem-loop RT primers significantly increase the specificity of reaction by reducing spurious priming with larger RNA species. According to reactions on synthetic mature and precursor miRNAs, such primers are at least 100 times better in discriminating between the mature miRNA and its precursor than corresponding linear primers. Second, stem-loop RT primers better discriminate between similar miRNAs that differ by only two bases. Finally, and perhaps most importantly, the efficiency of priming reactions with stem-loop RT primers is significantly higher, enabling sensitive, specific, and accurate miRNA profiling of a single cell.

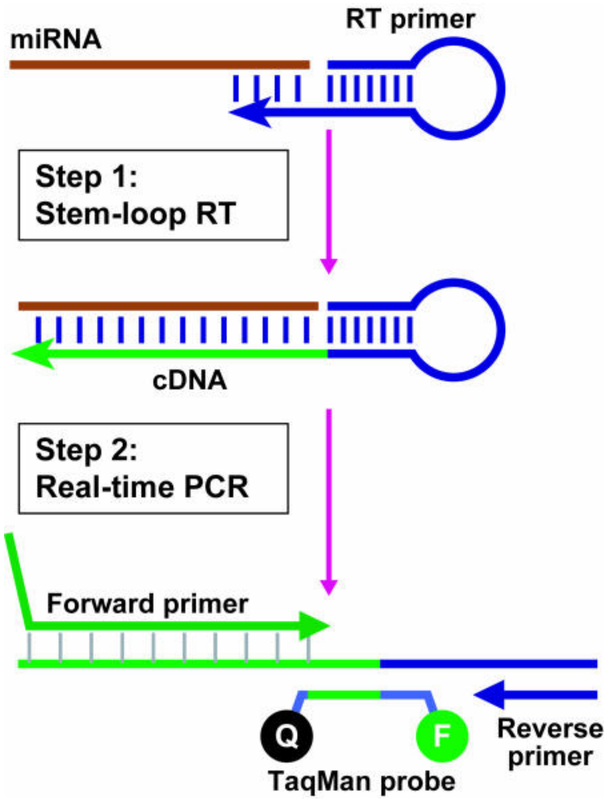

**FIGURE 1.** Schematic description of TaqMan-based real-time quantification of miRNAs includes two steps: stem-loop RT and real-time PCR. Stem-loop RT primers bind to at the 3′ portion of miRNA molecules and are reverse transcribed with reverse transcriptase. Then, the RT product is quantified using conventional TaqMan PCR that includes miRNA-specific forward primer, reverse primer, and dye-labeled TaqMan probes. The purpose of tailed forward primer at 5′ is to increase its melting Tm, depending on the sequence composition of miRNA molecules. An intermediate step of pre-PCR amplification can be added in multiplex reactions (see explanation in the text). The figure is reproduced from Chen et al.[41].

Theoretically, both methods can be multiplexed by using a mix of primers specific for several (or many) miRNAs and, thus, employed for screening of differentially expressed miRNAs or relative abundance miRNA profiling in small samples. For such multiplexing, primer design becomes especially critical since overlap of the 3’ ends of any two primers in a multiplex reaction can cause serious primer-dimer problems for PCR. Lao et al.[42,43] recently investigated the parameters associated with multiplexing stem-loop real-time RT-PCR for miRNA and came to several important conclusions. First, the RT step can be multiplexed using a mix of reverse primers. Priming efficiency is a function of priming sequence length and is optimal when complementarity between miRNA and RT primer is 8 nt. Second, multiplexing pre-PCR amplification can further increase sensitivity and specificity of the procedure. This optional step of 14–18 PCR cycles is carried out with the multiplexed forward primers, containing 3’ sequences that correspond to the 5’ end of miRNAs and additional 5’ sequences zip coded to each miRNA. The last step of the procedure is an individual singleplex PCR with miRNA-specific primers and TaqMan probe. Next, zip coding of all primers (reverse and forward) and TaqMan probe with sequences specific to each miRNA further improves reaction specificity as was tested in a procedure multiplexed for 330 miRNAs. This work demonstrated that relative abundance profiles of multiple miRNAs can be generated for most miRNAs from very small samples. However, it should be emphasized that multiplexing also boosts primer-primer interactions and may, therefore, lead to an increased amount of false-positives. It is recommended to apply the method to a moderate level of multiplexing, such as 48-plex, and to fine tune primer mixtures for particular studies.

We have recently used a two-step multiplex protocol with an omitted pre-PCR step to profile expression of miRNA in specific populations of cells isolated from human brain. We have used a multiplex of 400 RT primers (400 sets of miRNA RT primers), and sets of PCR primers and probes for 50 selected miRNAs of interest. For 48 out of 50 miRNAs, the accuracy of profiling by multiplexed RT-PCR has been validated by singleplex reactions (the results of this work to be published elsewhere). Kye et al.[44] recently used multiplex RT-PCR and detected multiple miRNAs in laser-captured neurites of cultured neurons. Therefore, we believe that multiplexing RT-PCR is an excellent method for medium-throughput miRNA screening or profiling in small samples when only picograms or nanograms of total RNA are available. It can be used for profiling specific cell populations, cell layers, and, potentially, for single cell[45] or even isolated cell compartments or ribonucleoprotein complexes. Nevertheless, the obtained data should be treated as microarray data are treated, i.e., normalized to expression of housekeeping genes and validated by miRNA-specific reaction like singleplex real-time PCR.

Since it is difficult to measure concentrations of RNA isolated from a few cells and, therefore, to control an equal input of RNA in the RT reaction, normalization is crucial. Housekeeping miRNAs are unknown; therefore, genes used for normalization (mRNAs, miRNAs, or snoRNAs) should be chosen carefully for each individual study. For example, we found several snoRNAs (snoR-2, snoR-13, and snoR-14) uniformly expressed in diverse cell populations isolated from human brain and used them for normalization of a corresponding miRNA expression dataset obtained by qRT-PCR.

## SMALL RNA PROFILING BY CLONING METHODS

The methods described above are extremely useful for profiling known miRNAs. Obviously, they depend on prior knowledge of the miRNA sequences. We do not know, however, how many additional small RNA molecules remain to be discovered. To characterize the miRNA repertoire of a particular cell type, tissue, or organ fully, a discovery approach must be taken. Bioinformatic approaches have been instrumental in the identification of novel miRNAs from genomic sequences[46,47,48,49]. However, these approaches rely on conservation and predicted hairpin structure. That this level of stringency may obscure miRNAs with important function has already been demonstrated[50]. In addition, computational approaches have identified many miRNAs that have not yet been detected experimentally. One reason that bioinformatically identified miRNAs may not have been observed experimentally is that these miRNAs are expressed in a temporally and spatially specific manner in complex organisms.

Most of the known miRNAs have been discovered by direct cloning approach. In some cases, an initial understanding of expression patterns has also been obtained by cloning. Many neuronal miRNAs, for example, have been cloned from brain, but not from other organs and, rationally, this suggests their role in brain development and/or function[51,52]. Besides identification of novel miRNAs, a cloning technique allows identification of other short RNA species with an as-yet-undescribed function. This precedent has been recently set by the discovery of ~30-mer short RNAs associated with testis-specific Miwi and Milli proteins[53,54]. Furthermore, a cloning approach can be used as a high-throughput semi-quantitative tool for expression analysis of small RNA molecules. The idea is that the relative cloning frequency of small RNAs represents a measure of their expression. Several studies have tested how well cloning frequencies reflect relative concentration within a sample. Generally, there is a high degree of correlation between the calculated number of copies per cell as measured by miRNA-specific hybridizations or RT-PCR techniques and relative cloning frequency of each of these miRNAs[35,55]. Nevertheless, the correlation is not perfect, which suggests that there is a systematic bias in the cloning protocols. The most likely source of this bias is the secondary structure of the different small RNAs that affect the adapter ligation efficiency. Therefore, a cloning approach should be regarded as an excellent, albeit very laborious, technique for semi-quantitative assessment of miRNA/small RNA expression.

An additional advantage of direct cloning is that it is the only method that enables identification of sequence variations, mutations, and the single nucleotide polymorphism associated with a sequence of mature miRNA. The frequency of sequence alteration is dependent on the specific nucleotide and its position with the miRNA, and is also tissue dependent[55]. The prevailing modifications are post-transcriptional, and caused by either 3' terminal A and U additions or A-to-I editing by adenosine deamination. Interestingly, the editing occurs four times more often in humans than in mice (overall 2.2% in humans and 0.5% in mice). Although the frequency of these events is relatively low, some evidence suggests their importance. First, single nucleotide polymorphism associated with mature miRNA can alter the processing of pri-miRNA and affect miRNA expression[56]. Second, at least one highly edited site identified in the middle of the 5' proximal half "seed" region of miR-376 can shift silencing to a different set of mRNA targets[37]. Since mammalian editing enzymes ADARs (adenosine deaminases acting on RNA) are widely expressed in the brain and their mRNA substrates have mainly been found in the CNS, it will not be too surprising to discover "functionally" edited regulatory small RNAs with a shifted spectrum of mRNA targets in the brain.

Cloning of small RNA is a multistep technique for which several different protocols have been established independently. The detailed description of the cloning procedure, as well as technical challenges and the possible solutions, has been reviewed intensely by Aravin and Tuschl[57]. The cloning procedure requires the presence of 5' phosphate and free 3' hydroxyl in the small RNA for sequential ligation of the 3' and 5' adapter oligonucleotides. Small RNA in the starting amount of 50–200 μg is extracted from a separating polyacrilamide gel and dephosphorylated for joining the 3' adapter by T4 RNA ligase 1 in the presence of ATP. Alternatively, a chemically adenylated adapter and truncated form of T4 RNA ligase 2 (Rnl2) allows elimination of the dephosphorylation step. After 5' adapter ligation, the product is gel purified and reverse transcribed, and the cDNA is PCR amplified using primers cognate to the ligated adapters. The concatamerized products of the PCR reaction are then cloned and sequenced.

In order to classify cloned small RNAs, their sequences are first mapped to a genome. About half of the clones usually represent small RNA molecules, mainly miRNAs, which should be distinguished from the degradation products of abundant larger RNA species (rRNA, tRNA, snoRNA, snRNA, and mRNA). Typical miRNA has the following characteristics: (1) its length distribution is narrow and peaks between 21 and 23 nt; (2) its precursor forms a hairpin structure, therefore, the genomic sequencing flanking a miRNA sequence contains a highly complementary 20- to 30-nt segment; (3) in most cases, pre-miRNA processing results in asymmetric strand accumulation; (4) a miRNA 5' end is precisely processed and most often uridine; (5) a 3' end is usually variable and, at low frequency, can be post-transcriptionally modified by addition of adenosine or uridine; (6) mature miRNA, and often pre-miRNA sequences are often conserved in closely related species. In most cases, miRNAs are originated from not-repetitive genomic sequences. Interestingly, infrequent repeat-associated miRNAs do not preserve the processing strand asymmetry between sequence-related precursors[55]. These sequence features help to identify and classify novel miRNAs cloned from a pool of small RNA molecules.

Neilson et al.[35] optimized the procedure by cloning directly from the short RNA fraction eluted from Ambion's miRvana columns. This protocol, with a reduced number of gel isolation steps and increased PCR amplification, enables cloning of short RNAs from low nanogram amounts of total RNA and, therefore, allows identification of novel small RNA species from specific cell populations and immunoprecipitated mRNP complexes.

Recent technological advances may take the discovery of small RNA molecules to a new level. Developed by the 454 Life Sciences Co., the massively parallel sequencing system technology (MPSS) allows quantitative identification of millions of small RNAs in a single reaction without prior knowledge of their sequences and, therefore, makes it possible to identify nearly all of the small RNAs in a sample. The focus of this technology is an emulsion-based method to isolate and amplify clonal DNA fragments on microbeads *in vitro*, and a fabricated substrate and instrument that performs pyrophosphate-based sequencing ("pyrosequencing") in picoliter-sized wells[58]. Using this sequencing platform, Berezikov et al.[59] obtained ~400,000 reads from small RNA libraries prepared from the human and chimpanzee brain. Computational analysis of these sequences identified 447 new, previously unknown, miRNAs.

Overall, these new miRNAs were expressed at low levels, with only a few miRNAs represented by more than one read. Although such single reads may be caused by sequencing errors, sequence analysis along with genomic location and conservation, as well as their identification in different libraries, strongly suggest that they represent real miRNAs.

Of course, the physiological importance of miRNAs expressed at low levels remains to be investigated. Nevertheless, it is tempting to speculate that the expression of some of these molecules may be boosted in specific physiological conditions or disease and, thus, provide a background for development of new miRNA regulatory pathways. In addition, many of these low-abundant miRNAs are not conserved beyond primates, indicating their recent origin. These data suggest that evolution of miRNAs is an ongoing process and that emerging miRNAs may contribute to the diversity of cellular programs, and, therefore, to ongoing evolution of the most complex animal organ – the human brain.

## ACKNOWLEDGMENTS

I thank my laboratory members for insightful discussions. We are grateful to Caifu Chen from Applied Biosystems for sharing reagents. The work in the lab is supported by grants from the National Cancer Institute (CA116141), Brain Tumor Society, and Eleanor and Miles Shore Harvard Medical School Scholarship.
